# The success of regorafenib in hepatocellular carcinoma in a world of failures. Learnings for future developments

**DOI:** 10.18632/oncotarget.22382

**Published:** 2017-11-11

**Authors:** Maria Reig, Álvaro Díaz-González, Jordi Bruix

**Affiliations:** Jordi Bruix: Barcelona Clinic Liver Cancer Group, Liver Unit, Hospital Clinic Barcelona, IDIBAPS, University of Barcelona, Barcelona, Spain; Centro de Investigación Biomédica en Red de Enfermedades Hepáticas y Digestivas, University of Barcelona, Barcelona, Spain

**Keywords:** hepatocellular carcinoma, sorafenib, trial design, systemic treatment, new drugs

The story of systemic therapy for hepatocellular carcinoma (HCC) resembles a battlefield with only few survivors as a result of a positive outcome. Only sorafenib in first line [1] and regorafenib [2] in second line have demonstrated a survival benefit versus placebo. In 2017 the non-inferiority data for lenvatinib *vs* sorafenib in 1^st^ line were presented [3]. In between and afterwards, the list of failed agents include: sunitinib, linifanib, brivanib, sorafenib-erlotinib or doxorubicin-sorafenib combinations, everolimus, ADI-PEG (NCT01287585) and doxorubicin-Transdrug (NCT01655693) [4]. This has primed a kind of rollercoaster feeling when trying to predict the potential success of new agents. All tyrosine kinases inhibitors were expected to be positive after sorafenib but failures induced a pessimistic state. We are back now to an optimistic expectation about the potential of immunotherapy. While the future is impossible to be predicted, it is necessary to frame the challenges in the evaluation of new agents and the flaws that trial design may face if no learning is extracted from the available data.

Trial design is not written in stone and has specific issues in - definition and stratification of target population, - outcome assumptions for sample size calculation, and - proper registration of evolutionary events and follow-up decisions that may confound the main end-point. Thereby, treatment beyond progression needs to be properly registered to avoid a misleading survival analysis. Double-blind trials prevent the subjective decision to interrupt treatment. In open label trials, investigators may prime early or late treatment interruption according to their perceived safety and benefit of the drugs. If cross-over is allowed, the risk of flaw is further increased.

Characterization of the target population is a major aspect. Most HCC patients present underlying cirrhosis. If decompensated (jaundice, ascites, encephalopathy), the competing risk due to death related to cirrhosis may prevent the recognition of a survival benefit of the agent tested. Thus, Child-Pugh A stage is further refined by the absence of ascites and upper cut-offs in bilirubin and albumin concentration. Patients with major cancer related symptoms are also to be excluded and the common approach is to accept just PS 0-1. After the broad definition of liver function, it is worth to stratify patients according to their evolutionary stage and expected prognosis. The BCLC [4] intermediate and advanced stage provide a rough survival prediction, but the optimal is to use individual parameters, such as vascular invasion, extrahepatic spread, presence of cancer symptoms and alpha-fetoprotein. In addition, some interventions provide higher benefit in some patients. Hepatitis C virus patients and patients without extrahepatic spread benefit more from sorafenib [4]. Hence, if sorafenib is the comparator, these parameters should be considered at least in the final analysis. Finally, the path of care is not homogeneous over the world. Asian patients enter clinical trials at a more advanced stage and this explains the common finding of their worse outcome [4]. Thus, stratification may include geographic location.

Novel concepts further complicate the situation. Pattern of progression is a major predictor of outcome [2, 5, 6]. While development of new small intrahepatic nodules does not have an independent impact in prognosis, new vascular invasion or new extrahepatic spread predict a poorer survival. Similarly, patients intolerant to sorafenib have a better survival than those that interrupt because of progression [6]. At the same time, those that have developed dermatologic adverse events under sorafenib have a more indolent disease with better survival [7, 8].

When a better understanding of the molecular profile of HCC is shown to correlate with better or worse outcome, such profiling will be taken into account and used to enrich trials according to the abnormality to be targeted. Unfortunately, none of the available molecular classifications is able to predict prognosis when tested prospectively and abnormalities detected may not be druggable.

These comments expose the complexity of trial design, while also inform about how to inspect the results of any therapeutic trial. Otherwise, positive phase 3 trials or encouraging survival figures in phase 2 assays may prime an optimistic expectation may just be overtly biased. It is also possible that trial with agents with potential efficacy have failed because of a faulty design. Accordingly, while hope and optimism are worth, excessive wishful thinking because of phase 2 data may prevent a robust phase 3 assessment of an encouraging option. Faith of patients and physicians in the expected benefits of drugs with encouraging early data, will prime patient withdrawal from ongoing investigations and ultimately prevent the availability of robust data to apply evidence-based treatment to patients diagnosed with HCC. Researchers and official agencies should keep rigorous in this philosophy and secure that the willingness to promptly provide patients of today with some promising agent, prevents its proper evaluation for the patients of the future.

## References

[1] Llovet JM (2008). N Engl J Med.

[2] Bruix J (2017). Lancet.

[3] Cheng AL (2017). J Clin Oncol.

[4] Bruix J (2016). Gastroenterology.

[5] Reig M (2013). Hepatology.

[6] Iavarone M (2015). Hepatology.

[7] Reig M (2014). J Hepatol.

[8] Branco F (2017). Ann Hepatol.

